# Assessing the Direct Effects of the Ebola Outbreak on Life Expectancy in Liberia, Sierra Leone and Guinea

**DOI:** 10.1371/currents.outbreaks.01a99f8342b42a58d806d7d1749574ea

**Published:** 2015-02-19

**Authors:** Stephane Helleringer, Andrew Noymer

**Affiliations:** Department of population, family and reproductive health, Bloomberg School of Public Health, Johns Hopkins University, Baltimore, Maryland, USA; Population Health and Disease Prevention, Program in Public Health, University of California, Irvine, California, USA

**Keywords:** Demography, ebola, EBOV, Guinea, Liberia, life expectancy, Sierra Leone

## Abstract

Background: An EVD outbreak may reduce life expectancy directly (due to high mortality among EVD cases) and indirectly (e.g., due to lower utilization of healthcare and subsequent increases in non-EVD mortality). In this paper, we investigated the direct effects of EVD on life expectancy in Liberia, Sierra Leone and Guinea (LSLG thereafter). Methods: We used data on EVD cases and deaths published in situation reports by the World Health Organization (WHO), as well as data on the age of EVD cases reported from patient datasets. We used data on non-EVD mortality from the most recent life tables published prior to the EVD outbreak. We then formulated three scenarios based on hypotheses about a) the extent of under-reporting of EVD cases and b) the EVD case fatality ratio. For each scenario, we re-estimated the number of EVD deaths in LSLG and we applied standard life table techniques to calculate life expectancy. Results: In Liberia, possible reductions in life expectancy resulting from EVD deaths ranged from 1.63 year (low EVD scenario) to 5.56 years (high EVD scenario), whereas in Sierra Leone, possible life expectancy declines ranged from 1.38 to 5.10 years. In Guinea, the direct effects of EVD on life expectancy were more limited (<1.20 year). Conclusions: Our high EVD scenario suggests that, due to EVD deaths, life expectancy may have declined in Liberia and Sierra Leone to levels these two countries had not experienced since 2001-2003, i.e., approximately the end of their civil wars. The total effects of EVD on life expectancy may however be larger due to possible concomitant increases in non-EVD mortality during the outbreak.

## Introduction

Prior outbreaks of Ebola virus disease (EVD) have caused ≈1,600 deaths between 1976 and 2013,^1^^,^ 2 primarily in central African countries (e.g., DR Congo, Uganda, Gabon). However, more than 20,000 EVD cases were reported in 2014, due to an outbreak that originated in December 2013 in a remote area of Guinea in West Africa.^3^^,^ 4 ****This EVD outbreak has since spread to 9 countries, but 3 of these countries have been particularly affected: Liberia, Sierra Leone and Guinea (LSLG hereinafter).

Public health research on EVD in LSLG has focused on identifying 1) the modes of EVD transmission and related risk factors,^5^^,^ 6 ^,^ 7 ^,^ 8 and 2) the determinants of survival among EVD patients.^7^^,^ 8 ^,^ 9 ^,^ 10 ^,^ 11 Using this information, mathematical models have been devised to project the future course of the outbreak,^7^^,^ 12 ^,^ 13 ^,^ 14 identify possible EVD control scenarios in LSLG,^15^^,^ 16 ^,^ 17 and evaluate the potential for EVD spread to other countries.^18^^,^ 19

The impact of EVD on mortality at the population level has garnered less attention. It is unclear how much the EVD outbreak may have reduced life expectancy at birth (e_0_ hereinafter) in LSLG in 2014. e_0_ refers to the average number of years a hypothetical cohort of individuals would live, on average, if they were subjected for their entire life to the mortality conditions of a specific year. It is the most commonly used summary measure of mortality. In this paper, we assessed the effects of EVD on e_0_ in LSLG in 2014 using available data.

## Direct vs indirect effects of EVD on life expectancy

An EVD outbreak may impact e_0_ through several causal pathways. It may directly raise death rates due to high mortality among EVD cases. In prior outbreaks of EVD-Zaire (the species of EVD circulating in West Africa), the case fatality ratio (CFR hereinafter) ranged from 44% to 88%.^20^ It may also indirectly increase the risk of dying from non-EVD causes of deaths (e.g., Malaria), due for example to lower utilization of non-EVD health services or increased economic hardship. In Sierra Leone, the number of inpatient admissions at health facilities declined by 70% during the EVD outbreak.^22^ This is primarily due to large numbers of EVD deaths among healthcare workers (HCW),^21^ significant EVD-related increases in workload among surviving healthcare workers, and fear of EVD infection among potential patients. Reduced economic activity during the EVD outbreak may have limited the ability of households to pay for medical expenses, or to invest in preventive measures. It may also have accentuated food insecurity and malnutrition, thus increasing susceptibility to other infectious diseases.

## Data sources on non-EVD mortality in EVD-affected countries

Investigating these complex effects of an EVD outbreak on e_0_ requires information on the number of deaths by cause (EVD vs. non-EVD) and by age group, both before and during the outbreak. Unfortunately, data on deaths from non-EVD causes in LSLG are either outdated, incomplete or inaccurate. Civil registration is very low: the WHO country office in Sierra Leone, for example, reported that only 1 or 2% of the total number of deaths were registered in the country.^24^ Data from health facilities may not be representative of mortality trends in populations where a large proportion of deaths occur at home. Health facility data may also show spurious mortality declines when patients stop seeking healthcare due to fear of EVD infection, or when health workers stop recording clinical events due to EVD-related increases in workload.

The only available estimates of non-EVD mortality in LSLG document mortality prior to the EVD outbreak. The World Health Organization (WHO) and the Institute for Health Metrics and Evaluation (IHME) have each estimated the annual number of deaths in LSLG. They have also constructed country-specific life tables, i.e., tables which show the probability of surviving from one age group to the next and permit calculating e_0_. There are significant discrepancies in estimates of the number of deaths and e_0_ in LSLG however (see table 1): for example, the WHO estimate of e_0_ for Sierra Leone is 12 years lower than the IHME estimate. This is so because both the IHME and WHO life tables were derived calculated using different statistical models on the basis of very limited data (e.g., census and survey data).^25^^,^ 26 ^,^ 27 The most recent WHO life table refers to 2012, whereas IHME recently produced a life table for 2013.

This paucity of high-quality real-time data on non-EVD mortality in LSLG has important consequences for measurements of the impact of EVD on e_0_. First, it implies that is not currently possible to measure the indirect effects of the EVD outbreak on e_0 _(i.e., e_0_ reductions due to lower healthcare utilization or increased economic hardship). Instead, *in this paper, we focus on measuring the direct effects of EVD on e_0_, i.e., reductions in e_0_ resulting solely from the high mortality of EVD cases.* Second, assessments of these direct effects of EVD on e_0 _ will be affected by uncertainty about pre-outbreak levels of e_0__._

## Surveillance of EVD transmission and mortality

Estimates of the direct effects of EVD deaths on e_o_ will also be affected by uncertainty about the extent of the EVD outbreak in LSLG. EVD cases are first identified during clinical care and/or contact tracing, i.e., the process of notifying individuals who have come in contact with someone infected with EVD about their exposure.^28^ Disease outcomes (e.g., deaths) are recorded during patient follow-up, then they are tallied and reported to the ministries of health (MoH) of LSLG. Ultimately, the WHO and the MoH compile these data^29^ and publish counts of EVD cases and deaths every few days in situation reports.

EVD cases are classified in 3 categories: confirmed, probable and suspected.^7^ Confirmed cases require a positive laboratory result (e.g., through reverse-transcriptase polymerase chain reaction, RT-PCR). Suspected cases are individuals with sudden onset of high fever and prior contact with a suspected, probable or confirmed EVD case or with a dead/sick animal. Suspected cases also include individuals with multiple symptoms characteristic of EVD, and any individual who died suddenly of unexplained causes. Probable cases can be individuals with suspected EVD who have been examined by a clinician. They also include deceased individuals who had contact with a confirmed EVD case, but for whom no laboratory data are available. Suspected and probable cases may become confirmed when laboratory testing is done.

The accuracy of this EVD surveillance process has been contested. Not all cases are confirmed: both Liberia and Sierra Leone list significant numbers of EVD cases as “suspected” (see table 1), without further investigation. There may be delays in reporting EVD cases, and errors may also arise when health workers compute summary figures from individual case reports. Most importantly, some EVD cases may never be reported at all. The US CDC, for example, estimated that, at the end of August 2014, there may have been 2.5 times more EVD cases than were actually reported.^16^

The recording of EVD deaths suffers from additional difficulties, relative to the reporting of EVD cases. A significant proportion of reported EVD cases are lost to follow-up before an outcome (recovery, death) can be recorded. In clinical settings, high workloads may also prevent HCWs from documenting patient outcomes.The EVD surveillance system thus records significantly fewer deaths than expected. In Sierra Leone, for example, situation reports only record one EVD death for every 3 confirmed EVD cases, whereas data sets on patients with complete follow-up indicate that the CFR in the country is >70% (see table 1).^7^^,^ 8 ^,^ 29

**Table 1: Data sources on EVD and non-EVD mortality in Liberia, Sierra Leone and Guinea.** Notes: * The population size for each country are obtained from projections conducted by the UN population division and available at: http://esa.un.org/unpd/wpp/Excel-Data/Interpolated.htm; the figures used in this paper correspond to the "medium fertility" scenario devised by the UN population division ** The IHME counts of deaths and life tables for LSLG are available at: http://ghdx.healthdata.org/record/global-burden-disease-study-2013-gbd-2013-age-sex-specific-all-cause-and-cause-specific; *** The WHO counts of deaths and life tables for LSLG are available at: http://www.who.int/healthinfo/global_burden_disease/estimates/en/index1.html †The Liberian ministry of health does not report deaths separately by case definition since November 2014; ‡These figures are drawn from tables S10-S12 (confirmed + probable) and tables S14-S16 (confirmed + probable + suspected) of reference [29]. They concern the period from December 2013 to November, 25th 2014.

	Liberia	Sierra Leone	Guinea
**Population characteristics**			
**Population size in 2014 (projections)**			
UN World Population Prospects*	4,396,873	6,205,382	12,043,898
**Annual number of deaths (pre-outbreak estimates)**			
IHME (2013)**	32,695	61,508	110,013
WHO (2012)***	34,500	102,500	118,600
**Life expectancy (pre-outbreak estimates)**			
IHME (2013)**	63.1 years	57.7 years	60.2 years
WHO (2012)***	61.8 years	45.7 years	58.1 years
**EVD surveillance**			
**Cases (12/24/2014)**			
Confirmed	3,116	7,160	2,342
Probable	1,805	287	269
Suspected	3,198	1,756	19
Total (confirmed + probable)	4,921	7,447	2,611
Total (confirmed + probable + suspected)	8,115	9,203	2,630
**Reported EVD incidence (per 1,000 inhabitants)**			
Confirmed + probable cases	1.12	1.20	0.22
Confirmed + probable + suspected cases	1.85	1.48	0.22
**Deaths (12/31/2014)**			
Confirmed	--†	2,461	1,463
Probable	--†	208	276
Suspected	--†	158	0
Total (confirmed + probable)	--†	2,669	1,739
Total (confirmed + probable + suspected)	3,471	2,827	1,739
**Case fatality ratio**			
***Situation reports***			
Confirmed + probable cases	--†	0.36	0.66
Confirmed + probable + suspected cases	0.43	0.31	0.66
***EVD cases with complete follow-up and definitive outcome‡***			
Confirmed + probable cases			
All EVD cases****	0.71	0.73	0.66
Hospitalized EVD cases	0.62	0.60	0.59
Non-hospitalized EVD cases	0.84	0.91	1.00
Confirmed + probable + suspected cases			
All EVD cases	0.75	0.79	0.66
Hospitalized EVD cases	0.64	0.62	0.59
Non-hospitalized EVD cases	0.87	0.93	1.00

## General approach

Due to these data limitations, we thus investigated the following counterfactual question: how much would e_0_ have declined because of EVD deaths in 2014,* if the risk of dying from non-EVD causes remained at estimated pre-outbreak levels*? We proceeded in several steps:

1. We conducted an uncertainty analysis of the total number of EVD deaths having occurred in 2014. This analysis incorporated possible errors in EVD surveillance data. It produced a range of estimates for the numbers of EVD deaths in each country.
2. We obtained a standard age distribution of EVD deaths for each country by using published data on a) the distribution of EVD cases by age and b) variation in EVD case fatality ratios across age groups.
3. We combined this standard age distribution of EVD deaths with a) results from the uncertainty analysis (step 1) and b) estimates of the mid-year population of each country. In doing so, we produced multiple sets of EVD-specific death rates by age group.
4. We incorporated these sets of EVD-specific death rates into IHME and WHO life tables documenting pre-outbreak mortality in LSLG, and we measured the direct effects of the EVD outbreak by comparing measures of e_0_ with and without EVD deaths.

## How many EVD deaths were there in Liberia, Sierra Leone and Guinea in 2014?

We devised a simple model of the number of EVD deaths, which included two parameters: the extent of under-reporting of EVD cases and the CFR among EVD cases. We called C^T^ the true number of EVD cases and C^S^ the number of EVD cases reported through surveillance. Then, C^T^=β×C^S ^where β is the hypothesized ratio of true to reported EVD cases. When β<1, then the EVD surveillance system reports more cases than there actually are. When β>1, then some EVD cases are not reported by the EVD surveillance system. The true number of EVD deaths is D^T^, with:

\begin{equation*}D^{T}=\beta \times C^{S} \times CFR\end{equation*}

This model does not use reported counts of EVD deaths listed in table 1 because the recording of EVD deaths is affected by significant loss to follow-up and missing outcome data (see above). Instead we derived a range of estimates for the number of EVD deaths solely from a) reported counts of EVD cases, and b) hypotheses about the true levels of β and CFR.

Unfortunately, there are only limited empirical data about the extent of under-reporting of EVD cases in LSLG (i.e., β). Existing estimates of β have been obtained either from mathematical models^16^ or through phylogenetic studies of EVD transmission chains.^30^ There are more extensive data on the CFR during this outbreak. Among the subset of confirmed and probable EVD cases for whom a definitive outcome was recorded, the CFR is ≈66-73% in LSLG (see table 1).^7^^,^ 29 But these estimates only include EVD cases reported until the end of november.^29^ If survival of EVD cases improved after that date (e.g., due to establishment of additional EVD treatment units), then the true CFR in 2014 may be slightly lower. On the other hand, if cases lost to follow-up or with missing outcome data are more likely to have died (e.g., if they did not receive medical care) than cases for whom an outcome was recorded, then the true CFR in 2014 may be higher.

We thus analyzed 3 scenarios. In a "low EVD" scenario, we assumed that β=1 (i.e., all EVD cases were reported in 2014) and CFR=0.60. The CFR of this scenario (0.60) corresponds to CFRs observed among hospitalized EVD patients during this outbreak (table1).^7^^,^ 8 ^,^ 29 In a "medium EVD" scenario, we assumed that β=1.70, i.e., the true number of EVD cases in 2014 is 70% higher than reported counts, similar to estimates of the maximum extent of under-reporting obtained through phylogenetic studies.^30^ The CFR for this medium EVD scenario is 0.70, which corresponds approximately to estimates currently reported among EVD cases with complete follow-up. Finally, we considered a "high EVD" scenario, where β=2.5, i.e., similar to estimates of the extent of under-reporting obtained by the US CDC using mathematical models.^16^ The CFR in this high EVD scenario is 0.85; it corresponds to CFRs observed among non-hospitalized EVD cases (see table 1).

Since suspected cases may frequently include individuals who are not infected with EVD, we only considered confirmed and probable cases in our main analyses. We also assessed however how much larger the direct effects of EVD on e_0_ may be if some of the suspected EVD cases were “true” EVD cases (see appendix).

Using this approach, the estimated number of EVD deaths in 2014 in Liberia thus ranged from 2,928 (low EVD), to 5,979 (medium EVD) and 10,372 EVD deaths (high EVD). Similar figures for Sierra Leone were 4,468 (low), 9,122 (medium) and 15,824 (high). In Guinea, EVD surveillance data suggest that the CFR is at least 0.66 (see table 1). The lowest estimate of EVD deaths in 2014 was then 1,739 as indicated by situation reports, vs 3,198 in the medium EVD scenario and 5,548 in the high EVD scenario.

**Table 2: Estimated number of EVD deaths in affected countries (confirmed + probable cases):** Notes: in calculating the number of EVD deaths in 2014, we only considered confirmed and probable cases. The EVD-specific death rates are obtained by dividing the estimated number of EVD deaths by the population size obtained from the UN World Population Prospects (see table 1). ‡The low EVD scenario for Guinea corresponds to the number of deaths recorded by EVD surveillance (see table 1), since the CFR implied by surveillance data is > 0.60.

	Liberia		Sierra Leone		Guinea	
	**Number of EVD deaths**	**EVD-specific death rate (per 1,000)**	**Number of EVD deaths**	**EVD-specific death rate (per 1,000)**	**Number of EVD deaths**	**EVD-specific death rate (per 1,000)**
**Estimated EVD deaths in 2014**						
Low EVD scenario	2,928	0.67	4,468	0.72	1,739‡	0.14
Medium EVD scenario	5,979	1.36	9,122	1.47	3,198	0.27
High EVD scenario	10,372	2.36	15,824	2.55	5,548	0.46

## The age pattern of EVD mortality

For each scenario, we then distributed these estimated EVD deaths across age groups, according to a country-specific standard age pattern of EVD deaths (see appendix for calculation). We calculated age-specific EVD death rates by dividing the estimated number of EVD deaths in each age group by the mid-year population of that age group in LSLG in 2014. We called \begin{equation*}_{n}m^{^{EVD}}_{x}\end{equation*} the death rate from EVD at ages *x *to *x+n. *To assess the direct effects of EVD on e_0 _in each scenario, we added these sets of \begin{equation*}_{n}m_{x}^{^{EVD}}\end{equation*} to pre-outbreak age-specific death rates provided in the WHO and IHME life tables, and noted \begin{equation*}_{n}m_{x}^{{^{IHME}}}\end{equation*} and \begin{equation*}_{n}m_{x}^{^{WHO}}\end{equation*}respectively. We calculated the age-specific relative risk ratios associated with EVD deaths in each life table as (in the case of the WHO life table):

\begin{equation*}_{n}RR_{x}^{^{WHO}}= \frac{_{n}m_{x}^{^{WHO}}+_{n}m_{x}^{^{EVD}}}{_{n}m_{x}^{{^{WHO}}}}\end{equation*}

The largest increases in mortality associated with EVD occurred in Liberia in 2014 (figure 1), whereas EVD was associated with only minor increases in mortality rates in Guinea. In Sierra Leone, the IHME and WHO life tables yielded different assessments of the impact of EVD on age-specific mortality. According to the WHO life table (lower panel), age-specific mortality rates increased by at most 50% due to EVD, whereas according to the IHME life table (upper panel), EVD deaths were associated with increases in mortality rates greater than 100% in some age groups. In all countries, the largest increases in mortality occurred at adult ages, with significantly lower increases in mortality risk associated with EVD among children and older adults/elderlies.

**Impact of EVD deaths on age-specific death rates, by pre-outbreak life table and country:**
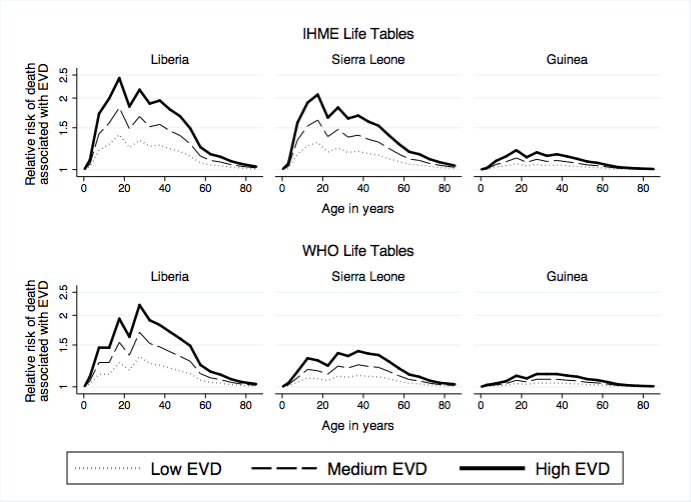
Notes: The relative risk ratios are calculated by dividing age-specific death rates in the presence of EVD deaths by age-specific death rates prior to the EVD outbreak. The y-axis is plotted on a logarithmic scale. EVD specific death rates were calculated by dividing the number of EVD deaths in 2014 in scenario, by estimates of the mid-year population for each country in 2014 obtained from UN projections. We used the mid-year population as an approximation of the number of person-years lived in each country in 2014. Unlike Liberia and Sierra Leone, the low EVD scenario for Guinea has CFR = 0.66. This is the CFR obtained from situation reports (see table 1).

## The direct effects of EVD deaths on life expectancy

We used standard life table techniques to calculate the direct effects of EVD on e_0_. For each scenario we considered 4 fictitious cohorts: two of these cohorts were subjected to the sets of pre-outbreak age-specific death rates calculated by IHME and WHO, i.e., \begin{equation*}_{n}m_{x}^{^{IHME}}\%0A\end{equation*} and \begin{equation*}_{n}m_{x}^{^{WHO}}\end{equation*} respectively; two other cohorts were subjected to similar death rates to which EVD-specific death rates had been added, i.e., \begin{equation*}_{n}m_{x}^{^{IHME}}+_{n}m_{x}^{^{EVD}}\end{equation*} and \begin{equation*}_{n}m_{x}^{^{WHO}}+_{n}m_{x}^{^{EVD}}\end{equation*}, respectively. For each cohort, we calculated the probabilities of dying between any two ages *x *and *x+n.* e_0_ is defined in each cohort as the total number of person-years lived by the cohort divided by the total number of cohort members. The direct effects of EVD deaths on e_0_ according to, say, the IHME life table are then defined as the difference in e_0_ between the cohort subjected to \begin{equation*}_{n}m_{x}^{^{IHME}}\%0A\end{equation*} and the cohort subjected to \begin{equation*}_{n}m_{x}^{^{IHME}} + _{n}m_{x}^{^{EVD}}\%0A\%0A\end{equation*}.

In Liberia (table 3), according to the IHME life table, the direct EVD effects on e_o_ in 2014 ranged from reductions of 1.63 years (low EVD) to 5.45 years (high EVD), vs. 1.94 years (low EVD) to 5.56 years (high EVD) according to the WHO life table. These direct effects of EVD deaths on e_0 _could be even larger in Liberia if some of the cases reported as “suspected” EVD cases were in fact true EVD cases. In that case, additional reductions in e_0_ of up to 1.5 years should be expected (see appendix, figure 2). In Sierra Leone, direct EVD effects on e_o_ ranged from reductions of 1.53 years (low EVD) to 5.10 years (high EVD) according to the IHME life table, vs. 1.38 (low EVD) to 3.77 years (high EVD) according to the WHO life table. The additional effects resulting from the inclusion of suspected cases would likely be limited in Sierra Leone (<0.5 year, see appendix figure 2). In Guinea, the direct effects of EVD lead to e_0 _reductions of less than 1.2 year according to both the IHME and WHO life tables.

**Table 3: Estimates of the direct effects of EVD deaths on life expectancy, by country and pre-outbreak life table:** All the figures listed in the table are in years. ‡For Guinea, the low EVD scenario corresponds to the situation where CFR = 0.66 since this is the value observed through EVD surveillance and reported in situation reports.

	Liberia				Sierra Leone				Guinea			
	**IHME Life table**		**WHO Life table**		**IHME Life table**		**WHO Life table**		**IHME Life table**		**WHO Life table**	
**EVD Scenarios**	2013 e_0 _	EVD effect	2012 e_0_	EVD effect	2013 e_0_	EVD effect	2012 e_0_	EVD effect	2013 e_0_	EVD effect	2012 e_0_	EVD effect
Low EVD	63.1	-1.63	61.8	-1.94	57.7	-1.53	45.7	-1.38	60.2	-0.30‡	58.1	-0.45‡
Medium EVD		-3.25		-3.48		-3.05		-2.39		-0.62		-0.75
High EVD		-5.45		-5.56		-5.10		-3.77		-1.07		-1.16

## Discussion

Whereas prior outbreaks of EVD in sub-Saharan countries have had limited impact on mortality at the population-level, the 2014 West African EVD outbreak likely caused significant declines in life expectancy in Liberia and Sierra Leone. EVD deaths in 2014 likely resulted in e_0_ reductions between 1.63 to 5.56 years in Liberia and between 1.38 to 5.10 years in Sierra Leone, depending on assumptions about the under-reporting and the mortality of EVD cases. Compared to IHME estimates of trends in life expectancy since 1990 in LSLG,^32^ life expectancy may thus have declined in Sierra Leone and Liberia to levels these two countries had not experienced since 2001-2003 (see appendix figure 3), i.e., the end of their respective civil wars. In Guinea, the direct effects of EVD deaths on e_0_ in 2014 were more limited, i.e., < 1.2 year.

These calculations present several important limitations however. First, we focused on analyzing 3 scenarios defined by varying levels of under-reporting of EVD cases and CFR. We did not seek to identify the most likely estimate of the direct effects of EVD on e_0_ through more complex statistical models (e.g., maximum likelihood methods). Such models will likely require further empirical investigations of the extent of under-reporting of EVD cases during this outbreak, as well as additional assessments of mortality among EVD cases.

Second, our 3 scenarios may not adequately capture the uncertainty associated with the number of EVD deaths in 2014 and their effects on e_0_. For example, in our high EVD scenario, we assumed that there were 2.5 times more EVD cases in 2014 than reported. This factor has been used in several other studies to account for undetected cases in the EVD outbreak.^33^^,^ 34 The extent of under-reporting may however have declined over time, particularly when the number of beds available in Ebola Treatment Units started to increase and/or when the capacity to conduct contact tracing was scaled-up. If so, the β parameter should be lower in our high EVD scenario. Similarly, the range of uncertainty associated with the CFR among EVD cases may be slightly broader than we hypothesized here. Several Ebola treatment units (ETU), for example, have reported CFR below 0.6 among their patients, thus suggesting that the value of the CFR we used in our low EVD scenario may be too high.^10^^,^ 11 These ETUs however employed non-standard treatment protocol (e.g., administration of intravenous fluids), which were not used in other ETUs. It is unlikely that such CFRs applied at the country level.

Third, we assumed that the age pattern of EVD infection was similar among reported and unreported EVD cases. It is however possible that the likelihood of case detection varied with age. For example, if younger EVD cases are more mobile than older EVD cases, they may be more likely to be missed by contact tracing teams. Similarly, since children experience more frequent episodes of other illness (e.g., Malaria) than adults, they may be less likely to be classified as suspected EVD cases during initial investigations. If the average age of unreported EVD cases is lower than the age of reported EVD cases, then the direct effects of EVD on e_0_ may be larger than we estimated here.

Fourth, due to data limitations, we assumed that the risk of EVD infection/death was fixed over relatively long age groups, e.g., 0-14 years old. Within that range, the CFR may however be much higher among infants and children under 5 than among adolescents aged 10-14 years old. If so, then the effects of EVD deaths on e_0 _may be slightly larger than estimated here.

Fifth, we did not investigate possible gender differences in the effects of EVD mortality on e_0_, also due to data limitations. Calculating e_0_ separately by gender indeed requires that data on age at EVD death are disaggregated between men and women. Unfortunately, WHO and MoH situation reports do not present cross-tabulations of the number of EVD cases by gender *and* age. We thus do not know whether women are affected by EVD at a younger age than men, for example.

Finally, our calculations do not capture the indirect effects of the EVD outbreak on e_0_. During an EVD outbreak, deaths from non-EVD causes may increase because health services are disrupted and/or because households experience economic hardship. Unfortunately, it is not currently possible to measure these indirect mortality effects of the EVD outbreak due to lack of up-to-date real-time data on mortality from non-EVD causes. Vital registration systems in the three most affected countries are indeed too incomplete to detect spikes in non-EVD mortality. Similarly, data on deaths from health facilities are selective and may misrepresent non-EVD mortality trends. Estimates of the indirect effects of the EVD outbreak on mortality from non-EVD causes of death will require conducting retrospective mortality surveys after the outbreak is over. Plans to address future EVD outbreaks should include strategies to collect real-time data on non-EVD mortality, in order to adequately respond to emerging concomitant health threats (e.g., increased Malaria mortality).

Our analyses thus only provide initial estimates of the impact of EVD on e_0_ in the most affected countries. They should be refined using more detailed statistical models, after more complete datasets on EVD and non-EVD mortality become available. Despite these limitations however, this work has important implications. It shows that, for the first time, an EVD outbreak likely resulted in large declines in life expectancy in affected countries. This unprecedented mortality impact at the population-level may prompt further investments in drug and vaccine research, and/or in health systems strengthening, to limit years of life lost to EVD.

## Figures and Tables

**Additional e0 impact resulting from EVD infection among suspected EVD cases, by country and EVD scenario.**
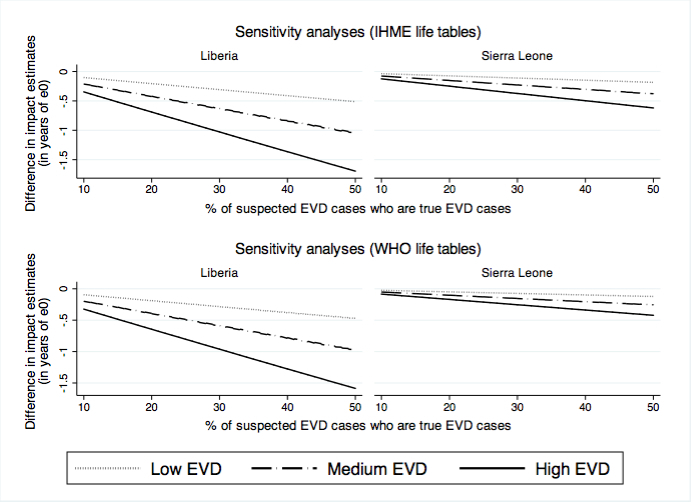
Notes: These figures represent the additional number of years of life expectancy that would be lost if a varying percentage of reported suspected EVD cases were actually infected with EVD. Guinea is not included in these analyses because very suspected cases are reported in that country (19/2730, see table 1). 3,198 suspected were reported in Liberia, vs. 1,870 in Sierra Leone.

**Time-series of life expectancy by year in Liberia and Sierra Leone (IHME estimates):**
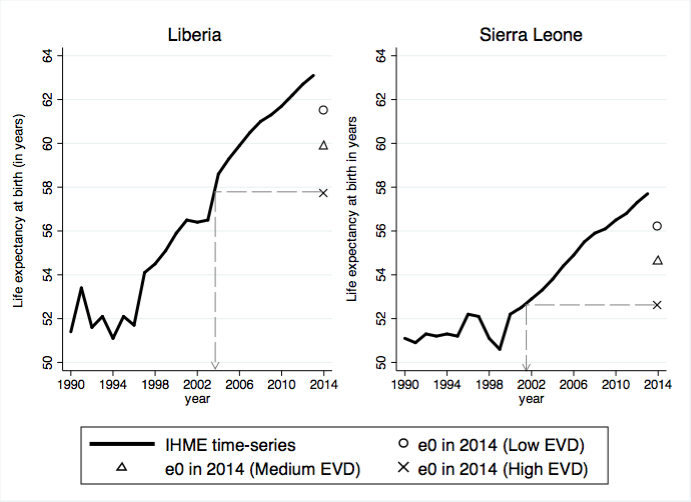
The time-series of e_0_ for 1990-2013 are available at http://ghdx.healthdata.org/record/global-burden-disease-study-2013-gbd-2013-age-sex-specific-all-cause-and-cause-specific.

## Appendix 1

### Data and Program

The data and program are available on figshare: http://figshare.com/account/projects/3396

### Accounting for illness duration and reporting delays in estimates of the number of EVD deaths in 2014

Since the average duration of illness ranges from 7.5 days to 10 days,^1^^,^ 2 ^,^ 7 ^,^ 29 ^,^ 31 the EVD cases who died in 2014 have, on average, become infected by December, 24th 2014 at the latest. We thus measured C^S ^as of 24th December 2014 (see table 1) to account for the time lag between onset of EVD symptoms and death.^31^  We also replicated our analysis using earlier reports of cumulative case counts to account for possibly longer duration of illness. For example, we used cumulative counts reported up to 20th December 2014. We also replicated our analysis using later reports of EVD case counts (e.g., up until January, 6th 2015) to account for delays in reporting of EVD cases. These analyses however yielded virtually similar results (i.e., ≈0.1 year difference in e_0 _estimates at most) as the analyses we report here. 

### Calculations of the standard age pattern of EVD deaths in each country

Calculating the impact of EVD deaths on e_0_ requires data on the age at death from EVD. Such data have not been directly reported, but we inferred a standard age distribution of EVD deaths in each country from data on a) age distributions of EVD cases, and b) age-related variations of the CFR. The information on the distribution of EVD cases by age group, as well as on variations of the CFR across age groups, was obtained from various tables and figures in reference 29 . Specifically, the age distribution of EVD cases was extracted from figure S11 of that reference, using a software tool to scrape data from *.pdf graphs (DigitizeIt). Age-specific estimates of the CFR are only available for broad age groups (0-14, 15-44, 45+). They were obtained from tables S10-S12 of reference 29 . We assumed that the CFR is constant within these age groups and applies to all nested age groups within corresponding life tables. 

To calculate the standard age pattern of EVD deaths in each country on the basis of this information, we first  distributed 1,000 hypothetical EVD cases across 5 and 10-years age groups according to age distributions of EVD cases in table A1. We then multiplied the number of EVD cases in each age group by the estimated CFR for each age group. Finally, we calculated the resulting percentage of EVD deaths in each age group. Input data and resulting standard age distributions of EVD deaths for each country are presented in table A1 below.

**Table A1: Calculated age pattern of EVD deaths:** The percentages in the table may not sum exactly to 1, due to rounding.

**Age** ** group**	**Liberia**			**Sierra Leone**			**Guinea**		
	% of EVD cases	CFR	% of EVD deaths	% of EVD cases	CFR	% of EVD deaths	% of EVD cases	CFR	% of EVD deaths
0-4	.0392	.632	.0350	.0423	.722	.0418	.0591	.710	.0636
5-14	.1177	.632	.1051	.1312	.722	.1305	.0894	.710	.0961
15-24	.1554	.690	.1516	.1663	.688	.1574	.1598	.604	.1470
25-34	.2274	.690	.2217	.2198	.688	.2081	.2212	.604	.2037
35-44	.2155	.690	.2103	.2022	.688	.1916	.2061	.604	.1898
45-54	.1333	.799	.1504	.1145	.827	.1301	.1174	.744	.1331
55-64	.0589	.799	.0665	.0671	.827	.0764	.0947	.744	.1076
65+	.0526	.799	.0593	.0565	.827	.0641	.0523	.744	.0590

Based on this age distribution of EVD deaths and the numbers of EVD deaths calculated in table 2, we obtained three sets of age-specific death rates for each country (one for each EVD scenario) and pre-outbreak life table. These sets of age-specific death rates are presented in figure 1 of the appendix below, by pre-outbreak life table and EVD scenario.

### Sensitivity analyses: the effects of suspected EVD cases

The direct effects of EVD deaths on e_0_may be larger if (some of) the cases recorded as "suspected" are in fact true EVD cases. We thus considered that a varying fraction (10-50%) of all suspected cases were in fact infected with EVD in LSLG. Then we calculated how many EVD deaths could be expected among this group under the low, medium and high EVD scenarios. We added these EVD deaths among suspected cases to the EVD deaths among confirmed and probable cases calculated above (see figure 1). Finally, we distributed these additional deaths across age groups and we recalculated e_0_.

The addition of suspected cases had limited impact on estimates of the direct effects of EVD on e_0_ in Sierra Leone (figure 2). At most EVD infection among suspected cases resulted in 0.5 years additional reduction in e_0_ in the high EVD scenario. In Liberia, on the other hand, the direct effects of EVD may be significantly larger than previously calculated if EVD infection was in fact common among suspected EVD cases. In the high EVD scenario (figure 2), high levels of EVD infection among suspected cases could result in additional reductions in e_0 _of more than 1.5 years.

### Comparison of study results vs. trends life expectancy in Liberia and Sierra Leone

We compared the results obtained in table 3 to historical annual estimates of e_0 _for 1990-2013 produced by IHME.^26^ Both Liberia and Sierra Leone have experienced stagnation in e_0_ during the 1990's. In Liberia, e_0_ is estimated to have started to increase around 1997, before plateauing between 2000 and 2003, i.e., at the time of the second Liberian civil war. Then e_0_ is estimated to have increased steadily in Liberia, from 56.5 years in 2003 to 63.1 years in 2012._ _In Sierra Leone, e_0  _stagnated between 1990 and 2001, i.e., during the country civil war, before increasing steadily from 52.5 years in 2001 to 57.7 years in 2013.The reduction in e_0_ resulting from EVD deaths in our high EVD scenario would foster e_0 _levels similar to those experienced in Liberia in 2003 and in Sierra Leone in 2001, according to IHME (see figure 3 below).  
